# Treatment of infra-bony periodontal defects using a collagen membrane and a bone substitute of equine origin – a pilot study

**DOI:** 10.1080/07853890.2021.1897404

**Published:** 2021-09-28

**Authors:** C. Izidoro, J. Lobato, M. Vilhena, M. Nemésio, L. Proença, J. Mendes, R. Alves

**Affiliations:** a Periodontology Department, Clinical Research Unit (CRU), Egas Moniz Cooperativa de Ensino Superior, Caparica, Portugal; b Centro de Investigação Interdisciplinar Egas Moniz (CiiEM), Egas Moniz Cooperativa de Ensino Superior, Caparica, Portugal

## Abstract

**Introduction:**

One of the main goals of periodontal treatment is the regeneration of lost tissues, in order to improve the prognosis of the involved teeth [1–3]. Guided tissue regeneration (GTR) involves the use of a barrier with or without the use of bone substitutes [4]. Given the great diversity and constant evolution of materials, it is necessary to maintain a permanent evaluation of their clinical effectiveness. This study intends to evaluate the success and predictability of GTR using a new membrane and bone substitute of equine origin (Heart® and Mix Granules^®^ – Bioteck SpA, Turin, Italy), in the treatment of infra-bony defects with two or three walls [5].

**Materials and methods:**

This study was approved by Egas Moniz Ethics Committee and all patients signed an informed consent form. Patients were selected among those referred to the Department of Periodontology/EMDC, with indication for surgical periodontal treatment, presence of 2–3-wall infra-bony defects, probing depth (PD) ≥ 5 mm, infra-bony component ≥4 mm deep (evaluated radiographically) and attached gingiva ≥2 mm. Exclusion criteria were: cases with PIaque Index (PI) >20%, smokers, extension of the defect to the furcation zone, mobility grade III, endodontic pathology, diabetes or other pathologies that interfere with bone remodelling and metabolism or contraindicate periodontal surgery. All surgeries were performed by the same operator. The clinicians involved in the evaluation of the results had no intervention in surgery. The following clinical and radiographic parameters were evaluated at one, three and six months after surgery: PI, Gingival Index (GI), Probing pocket depth (PPD), Bleeding on Probing (BoP); Gingival recession (GR), height of keratinised gingiva (KG) and radiographic angle of the defect (RAD). In addition, the presence of possible intra-surgical or post-operative complications was also evaluated. The assessment of the defect depth reduction was based on standardised periapical radiographs obtained through the use of an individualised device (parallelizer).

**Results:**

A total of six participants (83% females, 17% males), with a mean age of 61.6 (± 8.1) years, were enrolled in the study. The number of defect walls was: 3 walls (50%), 2 walls (33.3%) and 2/3 walls (16.7%). After six months, an average clinical attachment level gain of 3.3 (± 1.4) mm, mean radiographic bone filling of 72 (± 29%) and a BoP reduction of 66.6% were observed. RAD average was 21.3 (± 1.2°) and GR changed from 2.2 to 2.7 mm. Membrane exposure was observed in 33.3% of the cases, however, there were no cases of infection or severe pain.

**Discussion and conclusions:**

The use of collagen membranes and corticocancellous bone grafts of equine origin seems to be a viable alternative in the regeneration of infra-bony periodontal defects.
